# Review of phytochemical and nutritional characteristics and food applications of *Citrus* L. fruits

**DOI:** 10.3389/fnut.2022.968604

**Published:** 2022-07-18

**Authors:** Shuxun Liu, Ying Lou, Yixian Li, Jiaojiao Zhang, Ping Li, Baoru Yang, Qing Gu

**Affiliations:** ^1^Key Laboratory for Food Microbial Technology of Zhejiang Province, College of Food Science and Biotechnology, Zhejiang Gongshang University, Hangzhou, China; ^2^College of Food and Health, Zhejiang Agriculture and Forestry University, Hangzhou, China; ^3^Food Sciences, Department of Biochemistry, University of Turku, Turku, Finland

**Keywords:** citrus, phytochemicals, nutrients, health benefits, food applications

## Abstract

Since the dietary regimen rich in fruits is being widely recognized and encouraged, *Citrus* L. fruits have been growing in popularity worldwide due to their high amounts of health-promoting phytonutrients and bioactive compounds, such as flavonoids, phenolic acids, vitamins, carotenoids, pectins, and fatty acids. The diverse physicochemical properties and multiple utilization of citrus fruits in food industry are associated with their unique chemical compositions. Throughout the world, citrus has been used for producing various value-added and nutritionally enhanced products, including juices, wines, jams, canned citrus, and dried citrus. However, the current studies regarding the phytochemical and nutritional characteristics and food applications of citrus are scattered. This review systematically summarizes the existing bibliography on the chemical characteristics, functional and nutraceutical benefits, processing, and potential applications of citrus. A thorough understanding of this information may provide scientific guidance for better utilizing citrus as a functional fruit and benefit the extension of citrus value chain.

## Introduction

**Figure 1:**
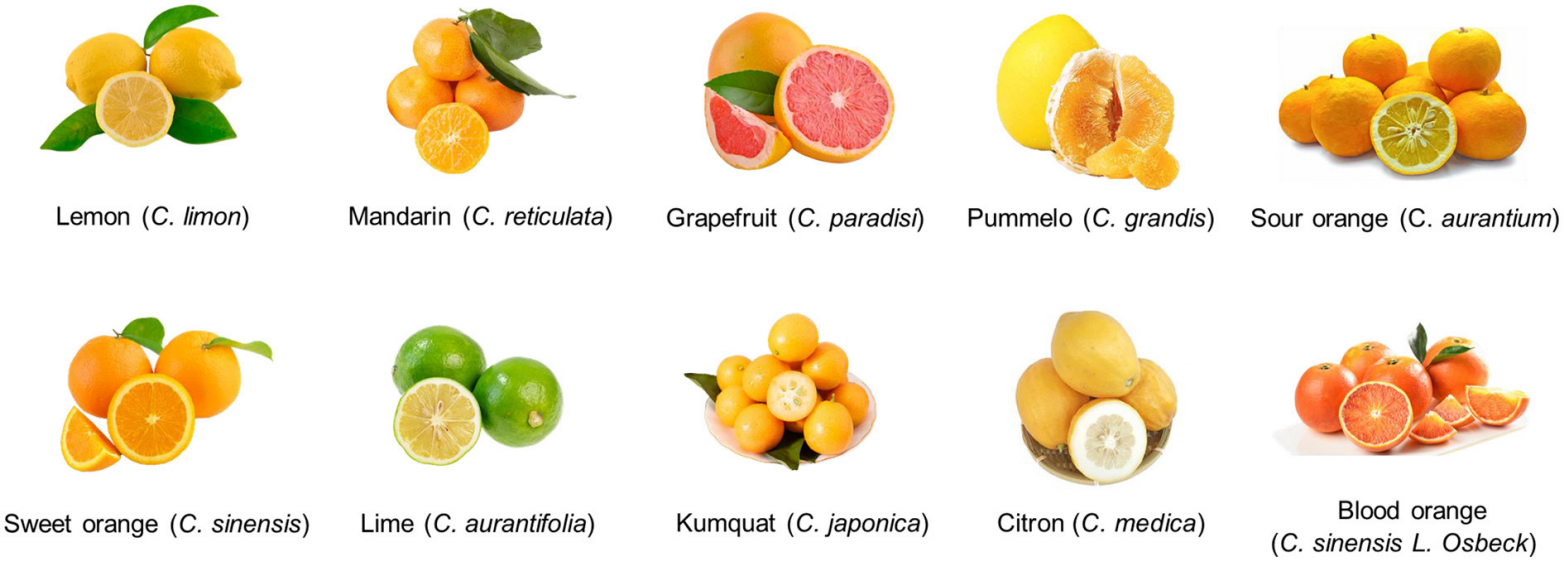
Phenotypes and genotypes of main citrus varieties and hybrids.

Citrus (genus *Citrus* L.), which belongs to the citrus genus of the *Rutaceae* family and *Aurantioidae* subfamily according to the botanical classification, is currently among the most economically important cultivated crops in terms of area and production values around the world. Citrus is believed to have originated in the Himalayan area of southwestern China, northeastern India, and northern Burma, and now has been diffused in more than 140 countries (1). According to statistics from the Food and Agriculture Organization of the United Nations (FAO), the total global production of citrus in 2019 was about 144 million tons, with a planting area of 9.89 million hectares, of which, the output and cultivation area of citrus in China reached 38 million tons and 2.88 million hectares, respectively, ranking first place worldwide. Sweet orange (*Citrus sinensis*), sour orange (*C. aurantium*), mandarin (*C. reticulata*), grapefruit (*C. paradisi*), pummelo (*C. grandis*), lemon (*C. limon*), citron (*C. medica*), lime (*C. aurantifolia*), kumquat (*C. japonica*), and hybrids are known as the most commercially important citrus species (Figure 1) (2).

Citrus fruits are common foodstuffs for human health thanks to their extremely high contents of health-promoting phytochemical and nutritional substances, such as multivitamins, pectins, carotenoids, fatty acids, and especially polyphenols (3). In recent years, along with the increasing public interest in plant antioxidants, the citrus fruits with high antioxidant ability associated with phytochemicals and nutrients are attracting more attention due to their potential health-promoting functions. The results of numerous epidemiological studies indicated a direct correlation between citrus fruit consumption and low risks of chronic diseases, such as cancer, cardiovascular diseases, and diabetes (4, 5). In addition, some curative components with pharmacological effects have also been found in citrus fruits and citrus by-products. Specifically, some phytochemicals in essential oils extracted from citrus peel have been demonstrated to have high free radical scavenging and anti-fungal activity; anti-pathogenic effects on healing sore throat, cough, earache, and vomiting have been demonstrated relating to citrus pulp and peel; and the extracts prepared using steam-distillation from citrus fruit and fruit/seeds have been used as a sedative and cardiac tonics, respectively (1, 6).

Short harvest time and shelf life are usually the flaws of fresh fruits and limit their availability and the possibility of enjoying the nutritional and functional properties all year round, as only a few days to months are generally the optimal time for fruit picking and consumption. Therefore, although most of the harvested citrus are freshly consumed, processing citrus into value-added products are important ways to extend the citrus industry chain and prolong the shelf life of citrus to several months or years, which is beneficial for the safe transportation of citrus products from the growing region of citrus to all over the world. However, currently, the actual conversion rate is extremely low in comparison with the colossal yield of citrus on the earth. For example, <5% of the total citrus yield in China is exploited annually. Even worse, a portion of the harvested citrus is lost because of the lack of adequate post-harvest management and processing infrastructures, which is a considerable economic loss for orchardists and farmers. Hence, processing of citrus into various products; such as citrus juices, citrus wines, citrus jams, canned citrus, and dried citrus is gaining attention by enriching the diversity of the citrus sector and thus minimizing postharvest losses.

To date, the information about the chemical composition, mainly including phytochemicals and nutrients, and the health-promoting properties and food applications of citrus is scattered. This study summarizes the current knowledge on the phytochemical and nutritional components in citrus. The health benefits of these compounds are discussed as well. Regarding the phytochemicals and nutrients, the composition, structure, and distribution, as well as bioactivities are priorly summarized. Thereafter, the utilization of citrus for the development of citrus products is reviewed. The review facilitates a better understanding of citrus composition and provides important references for future research and innovations to promote the production and utilization of citrus.

## Phytochemical profiles of citrus

### Flavonoids

**Figure 2:**
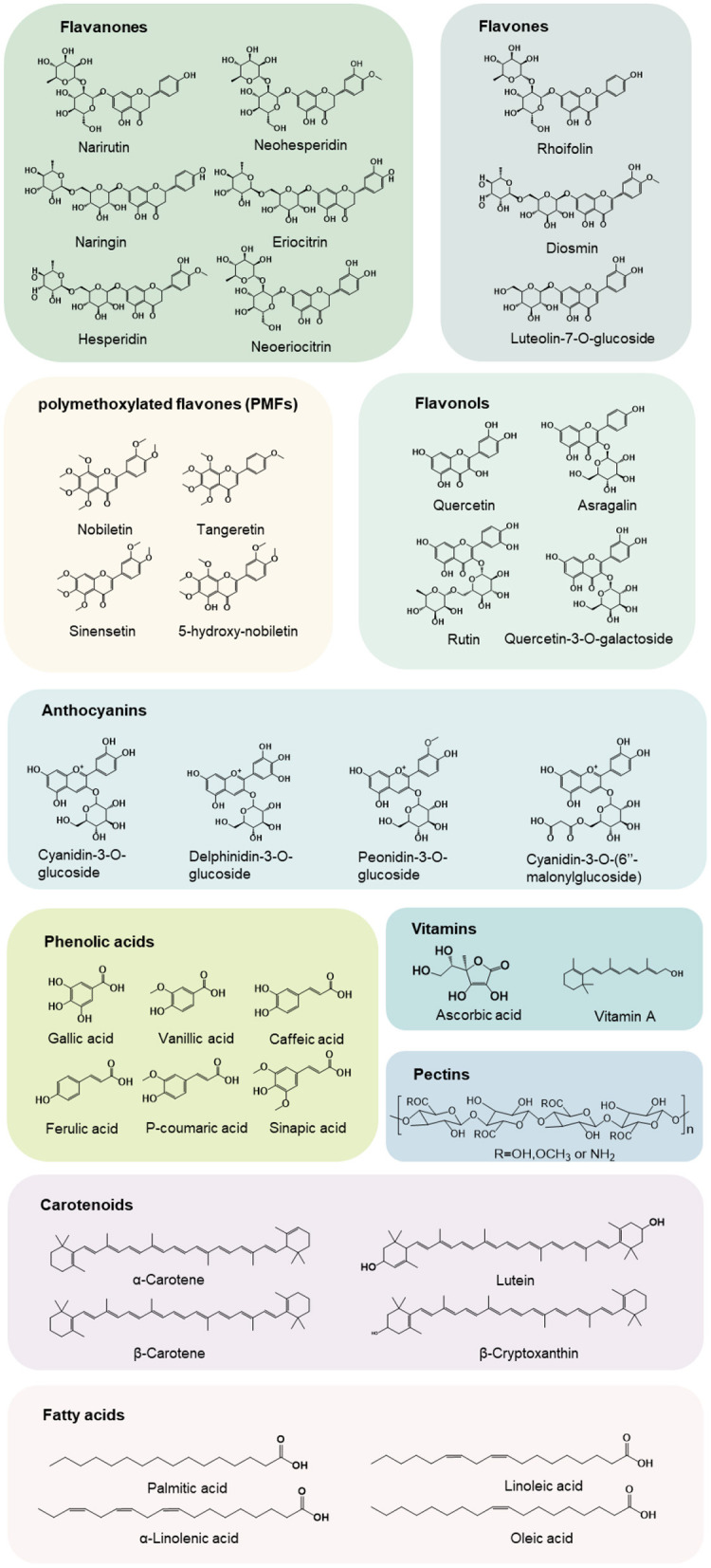
Chemical structures of major phytochemicals and nutrients in citrus.

As one the most abundant secondary metabolites, natural plant polyphenols have long been the subject of studies since they are essential for the protection of plants and exert functions of defense. To date, hundreds of polyphenols have been detected in citrus with flavonoids being the most important bioactive components with wide variety and distribution, and new polyphenols are being constantly discovered with the advancement in extraction and analytical methods (7–9). Flavonoids are naturally occurring low molecular weight phenolic compounds containing two aromatic rings (A and B) bound by a 3-carbon bridge (C6-C3-C6 structure) (Figure 2). Among the over 250 flavonoids have so far been identified in citrus (10), citrus flavonoids can be further structurally categorized into flavanones, flavones, flavonols, and anthocyanins (only in blood oranges), which are present in the form of aglycones or glycosides. Neohesperidoside and rutinoside are the most significant and common forms of flavonoids in citrus. Besides, hydroxylation and methylation are also frequently involved (11).

**Figure 3:**
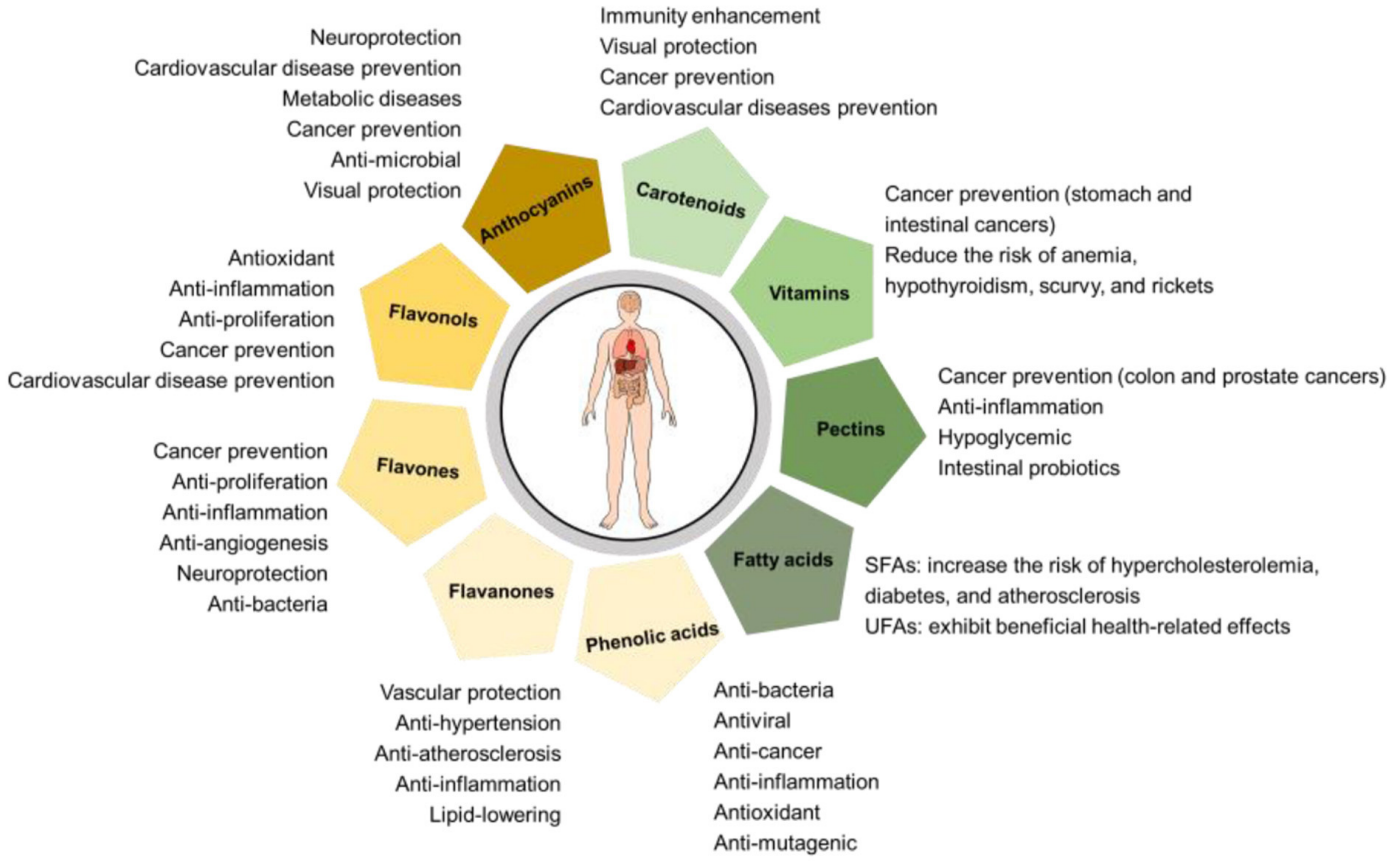
Potential health-promoting effects of phytochemical and nutritional compounds in citrus.

Flavonoids affect the quality of fruits in terms of taste, appearance, and bioactivities. For example, neohesperidosides, including naringin, neohesperidin, poncirin, and neoeriocitrin, consist of a flavanone with neohesperidose (rhamnosyl-a-1,2 glucose) and contribute the primary bitterness to citrus (12). Whereas rutinosides, such as hesperidin, narirutin, eriocitrin, and didymin, have a basic flavanone combined with a disaccharide residue of rutinose (ramnosyl-a-1,6 glucose) and are tasteless. Flavanones present in diglycoside form usually confer a typical taste of bitterness to citrus fruit (2). The accumulation of anthocyanins is the fundamental reason of pigmentation of blood oranges (13). With regard to the therapeutic and nutraceutical potentials, these bioactive ingredients help to reduce the risk of many chronic diseases (such as cardiovascular disease), with important nutritional health care functions (14). A large number of studies have demonstrated that citrus flavonoids can regulate the expression of inflammation-related factors, inhibit the adhesion of monocytes to endothelial cells, regulate cell migration and possess vascular protection, anti-hypertension, and anti-atherosclerosis effects (Figure 3) (15, 16). Nowadays, the prevalence of diabetes and obesity is rapidly increasing due to unhealthy dietary habits and a lack of daily physical exercise. Some *in vitro* studies have revealed that citrus flavonoids have the robust pharmacological properties of antidiabetic activity and anti-obesity action (17–19). For example, citrus flavonoid narirutin can block the digestive enzymes that hydrolyze polysaccharides into small absorbable fragments to regulate the blood glucose level in people with diabetes. Citrus flavonoids have been associated with reducing adiposity through the inhibitory effect on adipogenesis *via* the downregulation of genes associated with lipid metabolism and adipogenic transcription factors and the upregulation of certain lipolysis enzymes such as hormone-sensitive lipase (HSL) and AMP-activated protein kinase (AMPK).

The level and composition of flavonoids change with the development and maturation of citrus fruits and vary among different citrus species. A study on the flavonoid evolution during navel orange maturation found that the total flavonoid content continuously decreased during maturity, from 538 mg/100 g dry weight (DW) at the young fruit period to 69 mg/100 g DW at commercial maturity (20). Some researchers have evaluated the changes in phenolic profiles of lemon fruits during maturity and found that the total flavonoid content of lemon pulps ranged from 630 to 1460 mg/100 g fresh weight (FW) and the highest total flavonoid content occurred in April (21). A previous study accurately described the flavonoid profile of different citrus fruits using a new UPLC-PDA method, and the results indicated that the flavonoid contents in the fruits varied significantly among different citrus varieties cultivated in the same area, and different cultivated species were characterized by varying predominant flavonoid compounds; for example, naringin, erocitrin, and hesperidin are the most predominant components of pummelo, lemon, mandarin, and sweet orange, respectively (22). A comparative study on the flavonoid level in different citrus varieties reported that the total flavonoid concentrations in navel oranges (mean value: 95.3 mg/100 g FW), common oranges (mean value: 82.6 mg/100 g FW), and satsume oranges (mean value: 75.45 mg/100 g FW) were higher than that in clementine groups (mean value: 35.6 mg/100 g FW), sanguine groups (mean value: 56.9 mg/100 g FW), and hybrid groups (mean value: 56.4 mg/100 g FW) (23).

#### Flavanones

Flavanones are low in molecular weight, C6-C3-C6 parent nuclear structure consisting of 15 carbon atoms, and often contain multiple hydroxyl groups. Their flavonoid skeletons contain two aromatic rings (A and B rings), connected by an oxygen-containing heterocyclic ring consisting of three carbon atoms (C rings). Flavanones are the major polyphenols in citrus in comparison with other flavonoids and phenolic acids (24). Numerous epidemiological studies have reported the inverse relationships between citrus flavanones intake and the risks of cardiovascular diseases, hypertension, lipid-lowering, insulin-sensitization, atherosclerosis, and inflammation properties (Figure 3) (25, 26).

The compositions and contents of flavanones in citrus vary dramatically between different citrus varieties and fruit parts. For instance, the dominant flavanones in sweet oranges and mandarin both are tasteless hesperidin (15.25 mg/100 g FW) and bitter narirutin (2.33 mg/100 g FW), whereas in sour oranges the predominant flavanones are bitter neohesperidin (11.09 mg/100 g FW) and naringin (18.83 mg/100 g FW) (27). Pummelo is characterized by an abundance of naringin (218.65–783.91 mg/100 g FW), lemon is characterized by eriocitrin (9.46 mg/100 g FW) and hesperidin (15.78 mg/100 g FW) (22, 28). The concentration of naringin in grapefruits (654.35–987.17 mg/100 g FW) is comparatively higher than that in others (29, 30). Taking fruit parts into consideration, hesperidin is the dominant flavonoid in the pulps of lemons (74.3–181.6 mg/100 g FW), tangerine (167.5 mg/100 g FW), and sweet oranges (100.1 mg/100 g FW), and in the peels of mandarins (660.26–924.05 mg/100 g FW), sweet oranges (567.62–916.29 mg/100 g FW), and lemons (214.61–406.77 mg/100 g FW) (21, 22). The contents of eriocitrin reached 470.5 and 204.7 mg/100 g FW respectively, in the peel and pulp of lemon, which are much higher than those of the other citrus species (22).

The information regarding the distribution of flavanone in the products made from citrus is also considerable. HPLC-DAD-ESI-MS-MS separation allowed for the identification, for the first time, of narirutin 4'-O-glucoside, hesperidin, and narirutin in blood orange (Sanguinello and Tarocco) juices (31). Six flavanones of narirutin (39.91 mg/L), naringin (2.23 mg/L), hesperidin (171.17 mg/L), neohesperidin (0.95 mg/L), didymin (6.07 mg/L), and apigenin (32.37 mg/L) have been accurately quantified in orange juices made from Turkish cv. Kozan, while yeast fermentation significantly reduced their levels ranging from 42 to 50% (32). Gattuso et al. (33) summarized the flavanone composition in different citrus juices and found that the total flavanone contents in the juices made from *C. sinensis, C. reticulata, C. clementina, C. limon, C. aurantifolia, C. paradisi, C. deliciosa*, and *C. aurantium* are 4.74–73.17, 1.11–58.55, 5.26–86.22, 5.51–80.1, 1.52–2.02, 9.61–83.35, 2.12, and 4.34 mg/100 mL, respectively. Furthermore, hesperidin is usually the predominant flavanone in all the juices other than *C. aurantium* juice with this compound being virtually absent.

#### Flavones

Flavones possess 2-phenyl-1,4-benzopyrone skeletons, with hydroxyl groups usually at the 5- and 7-position, and often also at the 4'- and/or 3'-position. Six major flavone aglycones that are frequently reported include sinensetin, nobiletin, tangeretin, apigenin, luteolin, and diosmetin (Figure 2). The types and substitution positions of flavone glycosides are more diverse than those of flavanones. Glycosides can be substituted at positions 5, 6, 7, and 8 in A ring and 3' and 4' in B ring. The glycosylation may take place in (1) one or more hydroxyl groups of the aglycone bounding to glycoses to form an acid-labile glycosidic O–C bond (namely flavone *C*-glycosides) or (2) direct linkage of the glycoses to the basic nucleus of the aglycone, *via* an acid-resistant C–C bond (namely flavone *O*-glycosides) (11). The types of glycosides include rutinoside, neohesperidoside, glucoside, rhamnoside, arabinoside, and xyloside.

A previous study has reported, for the first time, the presence of five *C*-glycosyl flavones (lucenin-2, vicenin-2, stellarin-2, lucenin-2 4'-methyl ether, and scoparin) and a flavone *O*-glycosides (chrysoeriol 7-*O*-neoesperidoside) in blood orange juices (34). Abad-García et al. (35) have developed a method using HPLC-DAD coupled to ESI and triple quadrupole mass spectrometry to isolate different varieties of flavones from a series of citrus juices. The distinct flavones in the juices made from sweet orange, tangerine, lemon, and grapefruit are apigenin-7-*O*-rutinoside-4′-*O*-glucoside, luteolin-7-*O*-neohesperidoside-4′-*O*-glucoside, luteolin-6-*C*-glucoside, 6,8-di-*C*-acylhexosides of chrysoeriol and diosmetin, 6-*C*-and 8-*C*-glucoside-*O*-pentoside of apigenin, apigenin-6-*C*-hexoside-*O*-hexoside, and apigenin-8-*C*-hexoside-*O*-acylrhamnoside. Although the variety of flavones in citrus is diversified, the content of flavones only accounts for from trace to approximately 10% of total flavonoids (6, 14), which could be explained by the predisposed conversion from flavones into polymethoxylated flavones (PMFs) rather than being glycosylated (36, 37). Preclinical studies have demonstrated the promising physiological and pharmacological effects of citrus flavones, such as prevention and treatment of inflammation through the inhibition of regulatory enzymes or transcription factors that control mediators involved in inflammation (38), the antimicrobial effect on some Gram-negative and positive bacteria and *Saccharomyces cerevisiae* (39), and the anticancer activity *via* the suppression of cell growth and the promotion of cell death by regulating key molecular pathways related to cancer cell proliferation and immune cell function (Figure 3) (40, 41).

PMFs are unique constituents that almost exist exclusively in citrus and can thereby be used as biomarkers for citrus classification. PMFs exist in large amounts in the waxy constituents separated from the cold-pressed orange peel oil after long-term freezing treatment, while only a small amount is present in the fruit juice and pulp (42). PMFs, naturally occurring as free aglycones with at least two methoxy groups, have been reported in high concentrations in the peels of sweet, bitter, and mandarin oranges (29). Among the different citrus species, mandarins have the highest levels of PMFs, followed by sweet oranges, lemons, and pummelos (43). On the basis of the ESI-MS^*n*^ characteristics of PMFs and the results of EIC-MS/MS experiment, 32 PMFs have been screened from the complex extract of the peels of “Shatangju” mandarin (44). Nobiletin and tangeretin are common PMFs in citrus fruits, and supercritical carbon dioxide extraction using aqueous ethanol as co-solvent is considered the most environmentally friendly method for the extraction of these two PMFs from *C. depressa* Hayata peel (45). Many *in vitro* and *in vivo* studies have demonstrated anti-cancer, anti-proliferative, anti-inflammatory, anti-angiogenic, and neuroprotective properties of nobiletin and tangeretin (Figure 3) (46). However, these two PMFs are scarcely used as functional foods due to their low water solubility. Some studies have used debranched waxy maize starch *via* encapsulation to enhance the storage stability of tangeretin (47). Meanwhile, Sun et al. (48) have developed a method to encapsulate nobiletin using the cinnamaldehyde-modified whey protein stabilized microcapsules. The study indicated that the presence of cinnamaldehyde maintained or increased the bioaccessibility of nobiletin, regardless of emulsions or microcapsules.

#### Flavonols

Flavonols, the ancient and widespread class of polyphenolics, exhibit great antioxidant potential and are considered effective UV filters, thereby performing important functional roles during plant evolution (49). Flavonols represent the main fraction of bioactive compounds in plants with antioxidant, anti-inflammatory, and anti-proliferative capacities, having great health-promoting effects such as protection against cancer and cardiovascular disease (Figure 3) (50). Compared with flavone and flavanone aglycones, the main feature of flavonol aglycone is the existence of a hydroxyl group at C3 in the C-ring. Flavonol glycosides, especially *O*-glycoside, have the sugar moieties attached at position C7 of A-ring and position C3 of C-ring.

Kaempferol, quercetin, limocitrin, and isorhamnetin are the four main flavonols in citrus. The contents of flavonol in citrus juice generally are low in the range of 0.74–3.4 mg/100 mL (36). A comparative study suggested that the distribution of flavonols in citrus usually is variety dependent as the percentages of flavonols in the total polyphenol content are 1.0–3.5% in sweet orange juices, 0.3–9.5% in tangerine juices, 3.4–6.0% in lemon juices, and 0.1–0.2% in grapefruit juices (24). The accumulation of flavonols is divergent in citrus organs, as they are preferentially accumulated in leaves of most citrus varieties, while only a very small amount of flavonols is found in fruit tissues of a limited number of varieties such as lemon and lime (51).

#### Anthocyanins

Anthocyanins are water-soluble flavylium cation derivatives belonging to the flavonoid family involved in nature in several aspects of plant development and defense (52). Their unique structures in C rings are different from that of other flavonoids. In general, anthocyanin aglycones (namely anthocyanidins) are rarely found in nature due to their poor stability, while usually exist in the form connected with glycosides. According to the difference in the patterns of hydroxylation and methylations on the different positions of B ring of aglycones, approximately 25 anthocyanidins have been identified in nature. However, only six of them, including pelargonidin, cyanidin, delphinidin, peonidin, petunidin, and malvidin, linking with different glycoses, such as glucose, rhamnose, galactose, arabinose, xylose, and rutinose, are commonly found in plant-based food, accounting for about 95% of all anthocyanins (53). Apart from the chromogenic properties, anthocyanins are also important components of the human diet and, thanks to their radical-scavenging properties, therapeutic agents (54). The biological and pharmacological studies on these molecules have demonstrated their counteractive effects on oxidative stress and onset and progression of several non-communicable diseases (e.g., neurodegenerative, cardiovascular, metabolic diseases, and cancer), antimicrobial ability, and visual protective function (Figure 3) (55, 56).

Blood oranges, such as Tarocco, Moro, and Sanguinello, are typical varieties colored by anthocyanins that are present in both the rind and fruit juice vesicles. The content and composition of anthocyanins in blood oranges vary tremendously depending on variety, maturity, region of cultivation, and many other environmental factors. Cebadera-Miranda et al. (57) identified ten different anthocyanins, including seven cyanidin derivatives and three delphinidin derivatives, in blood oranges, and reported that cyanidin 3-(6"-malonylglucoside) and cyanidin 3-glucoside are the most abundant anthocyanins in both Sanguinello and Tarocco varieties. These two anthocyanins have also been dominantly found in Moro oranges (58). The total anthocyanin concentrations in Tarocco Ippolito (8.67–25.26 μg/100 g DW) and Sanguinelli (10.53–22.70 μg/100 g DW) generally are higher than that in Tarocco Rosso (6.95–12.12 μg/100 g DW) (57). Lo Piero (54) found that pigmentation levels varied significantly with cultivars of pigmented sweet oranges growing under even the same conditions. Specifically, the pigmentation level in OTA9 is highest, followed by Moro (high pigmentation), Tarocco (low to medium pigmentation), and Navelina (no pigmentation), in decreasing order. With regard to the environmental factor, cold treatment has been documented to extend Tarocco's shelf life and increase anthocyanin levels (59, 60). The anthocyanin accumulation in blood oranges during storage at low temperatures could be explained by the activation of enzymes involved in phenylpropanoid metabolism (61).

The accumulation of anthocyanins during plant development suggests that anthocyanin evolution is controlled and induced in favor of plants (54). Some studies have demonstrated that the accumulation of anthocyanins in blood oranges is closely correlated with fruit maturity. Cotroneo et al. (62) reported that the total anthocyanins of Moro orange are undetectable in the first two ripening stages (mid-to-late October to the beginning of November). However, their levels rise from the third and fourth stages (end of November to mid-December), remaining below 2 mg/100 g FW, and show a sharp increase starting from the sixth and reaching 22.2 mg/100 g FW in the seventh stage. The direct correlation between the accumulation of pigments and transcript levels for genes encoding chalcone synthase (CHS), anthocyanidin synthase (ANS), and UDP-glucose-flavonoid 3-*O*-glucosyltransferase (UFGT) was confirmed using quantitative real-time reverse transcriptase-PCR.

### Phenolic acids

Phenolic acids are phenols containing one carboxylic acid and can be structurally divided into hydroxycinnamic acids, which are characterized by C6–C3 structures, and hydroxybenzoic acids, which possess common C6–C1 structures. Phenolic acids are often combined with other substances (organic acids, flavones, cellulose, flavones, proteins, lignin, monosaccharides, etc.) in the form of ester bond, glycoside bond, ether bond, and rarely exist in a free state (63).

Although a fraction of phenolic acids can be produced through the metabolism of other kinds of polyphenols by microflora in colon of humans and animals, the majority of phenolic acids are supplemented by dietary sources (63). The dietary phenolic acids exist ubiquitously in plants, as citrus is one of the most important sources of these antioxidants. The health-promoting effects of phenolic acids have been well acknowledged. For example, phenolic acids possess much higher *in vitro* antioxidant activity than the well-known antioxidant vitamins and have various biological activities of antibacterial, antiviral, anticarcinogenic, anti-inflammatory, anti-mutagenic, and vasodilatory actions (Figure 3) (63, 64). These functions emphasize the potential utilization of phenolic acids in citrus in pharmaceutical industries.

Phenolic acid composition and content are different in diverse citrus fruits, which are mainly related to fruit variety, part, and ripeness. For example, some studies have found that the content of phenolic acid in citrus peel is much higher than that in citrus flesh, particularly in semi-mature stage (20). Hydroxycinnamic acids, such as ferulic acid, *p-*coumaric acid, caffeic acid, sinapic acid, and chlorogenic acid, are mainly found in various parts of citrus fruits, while hydroxybenzoic acids, such as vanillic acid, *p*-hydroxybenzoic acid, and gallic acid, are found in a small amount in citrus peels (65). Seven phenolic acids, including four hydroxycinnamic acids (caffeic, *p*-coumaric, sinapic, and ferulic acids) and three hydroxybenzoic acids (protocatechuic, *p*-hydroxybenzoic, and vanillic acids), have been determined by HPLC-PDA in the peels of *C. reticulata* (66). A similar profile of phenolic acids has also been detected in calamansi peel (67). *p*-Coumaric (24.68%) and ferulic (23.79%) acids are the most abundant phenolic acids in the peel extract of bitter orange (68), and gallic acid is the most abundant in the peels of several pomelo varieties (45). However, the major phenolic acid in Persian lime is *p-*coumaric acid (37). Ferulic acid was found as the predominant phenolic acid in the range of 31.6–63.7 mg/L in blood orange juices (6). An accordant result (37.7 mg/L) has been detected in juices made from Valencia late (69).

The remarkable effect of maturation on the evolution of phenolic acids in citrus has been well demonstrated. For example, Hou et al. (20) suggested that the concentrations of most phenolic acids in navel orange decreased during fruit maturation with exception of free fractions of sinapic acid and bound fractions of ferulic and caffeic acids. A study assessed the evolution of phenolic compounds in Eureka lemon fruits during ripening and found that *p-*coumaric acid and ferulic acid are among the major phenolic acids, and the maximum levels of phenolic acids are accumulated in the pulps of April fruits (26.94 mg/100 g FW) but in the peels of June fruits (76.93 mg/100 g FW) (21). A previous study evaluated the content of *p-*coumaric acid in Persian Lime over time and found that the concentration of this acid increased significantly from week 1 (0.98 mg/100 g FW) to week 5 (3.04 mg/100 g FW), then decreased drastically till week 7 (0.155 mg/100 g FW), and keep constant in the subsequent weeks (37).

### Carotenoids

Citrus carotenoids are a class of C40 isoprenoids, categorized into carotenes (hydrocarbonated carotenoids) and xanthophylls containing one or more oxygens. Carotenoids have special relevance to human nutrition and health-related aspects of immunity enhancement, eyes protection, and cancer and cardiovascular disease prevention (Figure 3) (10). As an important source of natural carotenoids, citrus carotenoids not only contribute nutritional value to citrus fruits, but also are responsible for the attractive color of yellow, orange, and red (10, 70). Carotenoids are naturally occurring fat-soluble pigments with antioxidant functions and some of them (provitamin A carotenoids, including α-carotene, β-carotene, and β-cryptoxanthin) can be converted into vitamin A (retinol) (71), thus make citrus become a valuable source to fulfill the recommended daily ingestion specifications.

Approximately 115 carotenoids have been detected in citrus species, including both carotene (α-carotene, β-carotene, and lycopene) and xanthophyll (β-cryptoxanthin, lutein, zeaxanthin, and violaxanthin) (71). The total content of carotenoids in citrus is located in the ranges of 2.5–50.1 mg/100 g FW (72). The content and composition of carotenoids in citrus are closely correlated with the types of citrus variety, maturity, and environmental conditions of cultivation and storage. A comparative study evaluating the carotenoid contents in different citrus food matrices (pulp and fresh juice) and their content in bioaccessible fraction and relative bioaccessibility of bioactive carotenoids in different varieties of two citrus species that are highly consumed worldwide, sweet orange and mandarins, suggested that the pulp of citrus fruits contains a similar or higher content of soluble bioactive carotenoids compared to fresh juice. Consequently, potential nutritional and health benefits are obtained by the consumption of pulp with respect to fresh juice (73). During ripening, due to the gradual conversion of chloroplasts to chromoplasts, the carotenoid contents in citrus fruits keep increasing with the degradation of chlorophyll (74). Besides, the genotype and environment conditions also impact the content of carotenoids significantly. A large number of studies on different cultivated citrus species have agreed on the accumulation of violaxanthin and β-cryptoxanthin can only be noted in mandarins and oranges in comparison with lemon, pummelo, and grapefruit (75–77). Moreover, the carotenoid composition of mandarins, oranges, and their hybrids could be distinguished by the concentration of β-cryptoxanthin (78). The study of Dhuique-Mayer (79) revealed the significant differences in carotenoid contents of citrus juices produced from fruits grown in Mediterranean, subtropical, and tropical countries. Furthermore, during juice processing, the applications of thermal or other stabilization treatments and the acidic condition stimulate the formation of carotenoid isomers and rearrangements of epoxy groups (73). With the aim to improve nutritional and functional values of citrus fruits in terms of carotenoid accumulation, storage at 12°C has been suggested as the feasible postharvest strategy (80).

Bioavailability, including absorption, metabolism, tissue distribution, and bioactivity, is a necessary prerequisite for assessing the role of a bioactive compound on human health and is strongly dependent on bioaccessibility. Due to the disturbance of poor solubility and chemical stability, and easy combination with food components, carotenoids are usually poor in human digestion. Some studies carried out using *in vitro* digestion models reported that the bioaccessibility of carotenoids could be improved by the presence of other dietary constituents, such as flavanones and pectin (3, 81).

## Nutritional compounds in citrus

### Vitamins

Vitamins are a general term for a series of organic micronutrients that are required by organisms for proper functioning. The deficiency of vitamins is a major public health issue worldwide and is closely related to numerous serious health problems, such as anemia, hypothyroidism, scurvy, and rickets. According to statistics, more than two billion people are suffering from vitamin deficiency and most of them belong to developing countries (82). Vitamins generally cannot be synthesized by organisms, and need to be obtained through diet and other means, making it possible to consume citrus, the source of abundant vitamins, as an important dietary vitamin uptake.

The vitamins in citrus can be divided into two main categories, namely fat-soluble vitamins (vitamin A and vitamin E, etc.,) and water-soluble vitamins (vitamin C and B-complex vitamins, such as vitamin B1, vitamin B2, and folate, etc.,). Among them, vitamin E and vitamin C are the most representative components in these two categories in citrus, respectively. In citrus fruits, vitamin A is the only fat-soluble vitamin that exists in an adequate quantity in the form of provitamin A carotenoids, with the carotenes and β-cryptoxanthin as the major vitamin A precursors (83). Vitamin C is widely considered the most important water-soluble antioxidant in citrus. This vitamin can prevent digestive tract cancers such as stomach and intestinal cancers, inhibit the formation of harmful substances such as free radicals and lipid peroxide in the body, and protect intra- and extra-cellular compounds (Figure 3) (84). Citrus juices are excellent sources of vitamin C and are widely available and commonly consumed beverages worldwide. The study of Rampersaud and Valim (85) has suggested that citrus juices, especially orange and pink grapefruit juices, were more vitamin C-dense than other commonly consumed juices.

Many factors affect vitamin content of citrus, including variety, maturity, breeding and storage conditions, and rootstocks. Cardeñosa et al. (86) assessed the effect of the citrus rootstocks on vitamin content and antioxidant activity of citrus fruits and found that the differences in antioxidant activity and related vitamin compounds are mainly dependent on the citrus rootstock, despite ripening stage had also some particular effects. However, in comparison with variety, harvesting season presented more remarkable impacts on vitamin contents and antioxidant activity of sweet oranges, as the highest values were found in the oranges harvested in January.

### Pectins

Pectins are complex polysaccharide polymer compounds widely found in fruits, roots, stems, and leaves of plants, with a chain-like molecular structure. The majority of pectins generally coexist with hemicellulose, lignin, cellulose, and other components, with the highest content being in fruit cell wall (87). The binding unit of pectins is D-galacturonic acid linked by 1,4-bonds to long chains, which usually exists in the methyl esterified state, and also includes L-arabinose, D-galactose, D-sorbose, L-rhamnose and other sugars in its main chain (88). According to the difference in structure, pectins can be divided into homogalacturonans, rhamnogalacturonan I, rhamnogalacturonan II, xylogalacturonan, apiogalacturonan, galactogalacturonan, galacturonogalacturonan, and arabinogalacturonan (89). Additionally, pectins can also be classified into high methoxy pectins and low methoxy pectins based on methoxylation degree (88).

Pectins are mostly extracted from fruit processing by-products, with the characteristics of low cost, simple operation, and avoidance of resource waste. Among them, citrus pectins are among the main source of commercial pectins. Citrus pectin is mainly found in the primary cell walls of citrus peels with the content accounting for 18–25% of the dry weight of peel (90). Citrus pectins have been used as good stabilizers and thickeners to improve the texture and quality of foods such as fruit and vegetable juices, jams, and jellies because of their outstanding thickening, stabilizing, gelling, and emulsifying properties (91). In order to meet people's wide extensive demand for healthy food, citrus pectins have been used as ideal fat alternatives in food processing, such as baked goods, salad dressings, ice creams, and other foods with high-fat levels (89). In addition, citrus pectins can act as degradable environmental protection films being used as substitutes for synthetic macromolecular polymers in food preservation, which can not only prolong the fresh-keeping period of foods but also is environmentally friendly (92).

Citrus pectins have been demonstrated important physiological functions and biological activities for human health, to name a few, including the prevention of colon and prostate cancers, anti-inflammation, hypoglycemic effects, and promotes the growth of beneficial bacteria in the gut (Figure 3) (45, 89). Citrus pectins are known to influence carotenoid bioaccessibility and absorption in humans. The structure of pectins, more specifically regarding their degree of methoxylation, favors carotenoid bioaccessibility but impairs the intestinal absorption of carotenoids from citrus concentrates (3).

### Fatty acids

Fatty acids are organic compounds formed by an aliphatic chain and a carboxylic group normally bounded with glycerol-forming acylglycerides (93). They are generally derived from triglycerides and phospholipids, and are important nutritional substances and metabolites in living organisms (94). In citrus fruits, fatty acids constitute the largest citrus lipid components and vary in saturation and configuration being grouped into saturated fatty acids (SFAs) and unsaturated fatty acids (UFAs), while the latter can be further divided into monounsaturated fatty acids and polyunsaturated fatty acids. Epidemiological studies have reported that SFAs increase the risk of hypercholesterolemia, diabetes, and atherosclerosis, whereas UFAs such as linolenic and linoleic acids, exhibit beneficial health-related effects (Figure 3) (94). In general, UFAs predominate over the SFAs in citrus and represent approximately 70% of total fatty acid content (TFA) (95). Data from USDA Food Data Central indicated that the total SFA in tangerines, oranges, limes, and grapefruits are 39, 15, 22, and 14 mg/100 g FW, respectively, while the total UFA are 125, 48, 74, and 37 mg/100 g FW, respectively. Matsuo et al.(96) found that the major fatty acids present in three cultivars of citrus natsudaidai peel are linoleic acid (C_18:2_, *cis*-9, 12, 42.8–48.5% of TFA), followed by palmitic acid (C_16:0_, 20.2–20.8% of TFA), α-linolenic acid (C_18:3_, *cis*-9, 12, 15, 12.1–14.6% of TFA) and oleic acid (C_18:1_, *cis*-9, 8.60–10.1% of TFA). An accordant result has been reported in the study comparing the fatty acid profiles of six citrus species breeding in South Korea (93). Eighteen fatty acids have been identified in five citrus juices (blood orange, sweet orange, lemon, bergamot, and bitter orange) and the mean concentration of fatty acids varies from 311.8 mg/L in blood orange juice to 678 mg/L in bitter orange juice (95).

## Food applications of citrus

**Figure 4:**
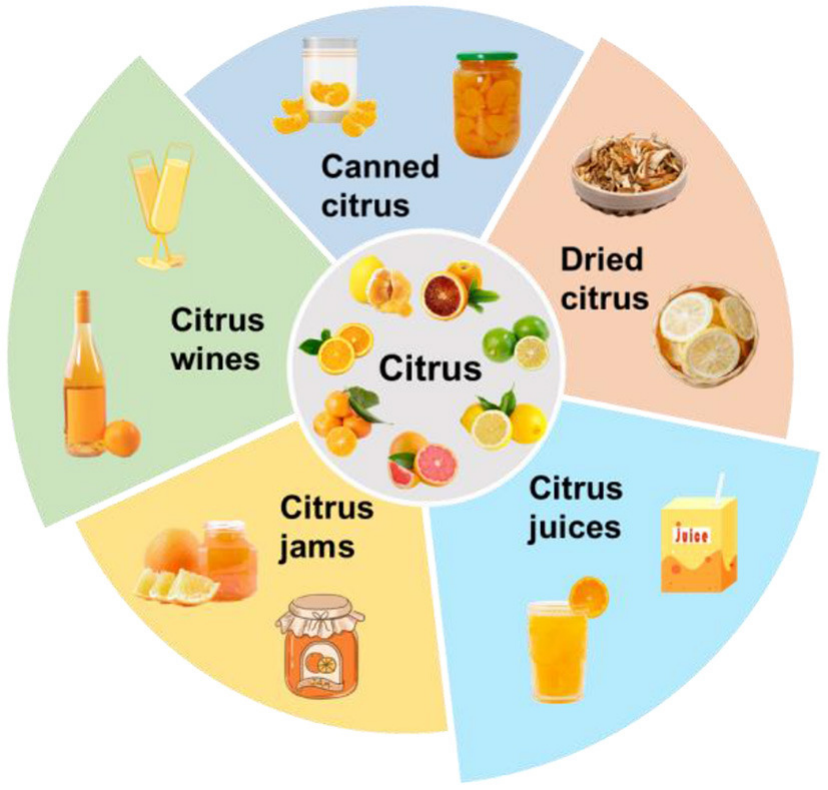
Current food applications of citrus.

Nowadays, the flaws of short harvest time and shelf life of fresh citrus limit their supply and availability. Moreover, some popular cultivated citrus growing areas are being expanded for economic benefits, thus causing an oversupply of citrus in the market. Hence, further preservation and processing of citrus are vital approaches to maintain the majority of nutritional and phytochemical values of citrus, and to make the best use of citrus resources. In this section, various applications on how to better utilize the citrus are provided to facilitate its food industrial application and thus extend its industrial chain. The food applications include citrus juices, citrus wines, citrus jams, canned fruits, and dried citrus (Figure 4).

### Citrus juices

Citrus juice is the most popular fruit juice with the largest trade volume worldwide, accounting for about 2/3 of the global juice market. However, the undesirable bitterness of citrus juice has always been the main problem faced by the citrus juice industry, which makes the product unacceptable. Studies have shown that the bitter taste of citrus after juicing is caused by two aspects, one is the bitter taste derived from the flavonoids and their derivatives in citrus fruit, and the other is the bitterness caused by the conversion of non-bitter precursors into bitter substances. For example, limonin A-ring lactone (LARL) can convert into limonoids by the enzymolysis of limonin D-ring lactone hydrolase (LDRLase) (97). Therefore, in recent years, researchers are focusing on debittering citrus juices to elevate their flavor and thus increase consumer acceptability. At present, an array of debitterness techniques, including physical, chemical, and biotechnology methods, have been developed. The mechanisms of bitterness reduction mainly include (1) removal of citrus parts that are rich in bitter components (such as peels segment membranes and seeds); (2) removal of bitter compounds in citrus (such as resins adsorption and ultrafiltration membrane filtration); (3) addition of bitter compound scavengers (such as cyclodextrin, neodiosmin, and neohesperidin dihydrochalcone); (4) enzymatic conversion of bitter compounds (such as naringinase, transeliminase, Acetyl-lyase, and limonoate dehydrogenase); (5) use of genetic engineering method to regulate the synthesis pathway of bitter compounds (98, 99).

Among the multifarious citrus juices on the market, the novel products using probiotics, such as *Lactobacillus fermentium, L. casei, L. plantarum*, and *L. pentosus*, for fermentation have received widespread attention (100–102). This kind of citrus juice makes it possible to integrate the characteristics and properties of citrus and fermentation strain, showing unique nutritional and functional values, such as anti-pathogenic bacteria, immune regulation, cancer prevention, and cardiovascular disease prevention, to name a few.

### Citrus wines

The development of citrus wine not only makes full use of citrus resources but also greatly increases the added-value of citrus, which is of great significance to promote the development of the citrus industry. Compared to wines, citrus wines are usually characterized by lower alcohol and mellow body. Moreover, citrus wine retains to a large extent the original flavor and functional components of citrus, including a variety of vitamins, polyphenols, pectins, carotenoids, and fatty acids. These nutrients and bioactive substances endow citrus wine with unique flavor and biological functions, such as antiaging, health care, nourishment, moistening the lung, nourishing the liver, and relieving tension (103).

In recent years, the research of citrus wine has attracted extensive interest, and numerous excellent results have been achieved at present. The flavor features of citrus wine are mainly determined by the substances derived from citrus fruit and that are produced by microbes during fermentation. Therefore, citrus varieties, fermentation technology, and microbial metabolism are the factors that have been placed more attention by researchers and fermentation practitioners for making citrus wine with premium quality. Nowadays, like citrus juice, citrus wine is also disturbed by strong bitterness. The debittering practices of citrus wine mainly include the selection of citrus varieties with low acidity, low content of bitter compounds, and high sugar content, the pretreatment of citrus juice in the early stage of fermentation, like that have been aforementioned in the section “Citrus Juices,” and optimization of final citrus wine, such as aging and fining (104). In addition, blended citrus wines fermented with citrus and other fruits or vegetables, such as apple, pineapple, carrot, and Chinese jujube are gaining ground in recent years (105, 106). The blended products could provide the advantages of multi-variety, multi-type, and multi-function that citrus single fermented wine lacks. Various indicators have demonstrated that the quality of wines made by multi-fruit blending has been significantly improved, which may provide a reference for the development of novel citrus wines.

### Canned citrus

Canned citrus is among the largest variety of canned foods in the world and plays an important role in the international canned trade (107). In recent years, canned citrus caters to modern and fast consumption mode and is welcomed by consumers. Thermal treatment is one of the main processes during the production of canned food. However, in the process of canning, sealing, and sterilization, high temperature will not only change the physical properties such as pulp chroma, hardness, and brittleness, but also accelerate the oxidation, decomposition, and loss of vitamins, carotenoids, flavonoids, reducing sugar and aroma substances, which will affect the color, flavor, and nutritional quality of canned citrus (108). Therefore, some new thermal methods, such as microwave, ohmic heating, and infrared radiation, and non-thermal protocols, such as pulsed electric fields, high-pressure processing, high-pressure carbon dioxide, sonication, and ultraviolet have been extensively applied in the development of canned citrus products. Compared to the conventional thermal treatments, these novel methods showed better preservation of the nutritional values and flavor of canned citrus (109).

The citrus canning processing water from the production of canned citrus is an important resource of pectin and sugar (110, 111). The wastewater mainly comes from scalding citrus, peeling and splitting, fruit sac transportation, acid-base treatment, classification, inspection and selection, sterilization, and so on. The citrus canning processing wastewater without treatment is detrimental to the water ecological environment, the growth and propagation of organisms, and even human life. Therefore, some methods, including physical method, chemical method, and biological method have been exploited to better utilize the citrus resource (110, 111).

### Citrus jams

Jam, made from fresh fruit with the addition of sugar, thickeners, and other excipients, is a kind of fruit processed product, which mainly aims to extend the preservation of fruits. Bright color, delicate taste, rich in nutrition, and unique flavor maintaining of fruits are the principal characteristics of jam. With the rise of fast-food culture, jam plays an important role in daily diet. Nowadays, citrus jams, such as orange jam and grapefruit jam, are widely consumed at breakfast and in dairy products, bakery products, and confectionery. Normally, in order to obtain better texture and retention, the sugar content of the traditional citrus jam is mostly between 50 and 70%, which results in a sweet and greasy taste. The existence of high sugar is harmful to human health, making the traditional citrus jam-making process unable to meet the requirements of contemporary consumers for healthy food. Hence, how to reduce the content of sugar in citrus jam is a topic of significance. The utilization of alternative natural sweeteners with the merits of non-cariogenicity and less glycemia, such as tagatose and isomaltulose, is considered an effective attempt (112). Moreover, to reduce the important losses of beneficial properties of citrus fruits resulting from the application of prolonged heat treatments during jam production, some novel protocols employing microwave heating technique and osmotic dehydration have been used as alternatives to the conventional methods (113, 114).

### Dried citrus

With the aim to extend the shelf-life of fruits, dehydration is usually a process that is widely employed for partial removal of water from high moisture fruits. In the case of citrus, both the pulp and peel of fruit, the pomace generated during juice production, and the juice are rich in polyphenols, vitamins, pectins, and other substances and can be dried to dehydrated products. However, undesirable drying protocols may result in dramatic degradations of phytochemicals and nutrients.

Numerous drying methods, such as freeze-drying, hot-air drying, vacuum drying, drum drying, microwave drying, and sun drying, have been applied to the dehydration of citrus and its by-products (107, 115). However, there are discriminations regarding the dehydration methods for different parts of citrus. Taking lemon as an example, the techniques of hot-air drying (45°C) and freeze-drying have been used for the dehydration of lemon fruits (116), hot-air drying (40, 50, and 60°C) and combined osmotic hot-air drying for lemon peels (117), and freeze-drying, hot-air drying (70, 90, and 110°C), and vacuum drying (70, 90, and 110°C) for lemon pomace aqueous extracts (118). Moreover, the applications of different drying techniques significantly impact the chemical composition of raw materials. For example, the flavonol concentration extracted from lemon fruit using hot-air drying is higher than that using freeze-drying, whereas the results of flavanone and flavone contents are contrary (116); freeze-dried lemon powder has a significantly lower phenolic content than the low-vacuum-dried lemon (119). In addition, drying temperature also influenced the evolution of polyphenols remarkably. For instance, the study of Papoutsis et al. (118) compared the vacuum dying temperatures of 70, 90, and 110°C on the recovery of flavonoids in lemon by-products and found that 70 and 90°C were the optimum rather than 110°C, while Ghanem et al. (120) found that the degradation rates of phenolics and flavonoids increased with increasing air drying temperature.

Drying of citrus juices to free-flowing powders that can be reconstituted with water to drinks of acceptable flavor meets the demand of convenience from consumers. However, citrus juices are much more difficult to dry than other liquid foods such as milk, due mainly to the high content of sugar mixtures. During the drying process, especially with high drying temperatures, citrus juice may become highly viscous, thermoplastic, and sticky masses. The commonly applied methods to overcome this problem are the addition of drying carriers, such as maltodextrins, low dextrose equivalent, β-cyclodextrin, and gum arabic, which acted to increase the glass transition temperature and sticky point of the concentrated juice (107). However, these carriers are usually expensive and undesirable in the composition of the final product. Hence, the application of suitable drying techniques could greatly protect citrus juice flavor, phytochemicals, and nutrients. Nowadays, the primary drying methods used in the dehydration of citrus juices include spray drying, drum drying, vacuum drying, foam mat drying, and freeze-drying (107).

## Conclusions and future perspectives

Citrus fruits are consumed in large quantities worldwide due to their attractive aromas and taste. The high abundance of phytochemicals, including flavonoids, phenolic acids, and carotenoids, and nutrients, including vitamins, pectins, and fatty acids, characterize citrus and citrus products' distinct flavor and various therapeutic and nutraceutical potentials, such as antioxidant, anti-inflammatory, antimicrobial, anticancer, neuroprotective effect, and other biological properties. The composition and content of phytochemical and nutritional compounds in citrus vary dramatically among varieties, fruit parts, maturity, region of cultivation, and many other environmental factors. The developments of value-added citrus products, including citrus juices, citrus wines, citrus jams, canned citrus, and dried citrus, can partly overcome the defects of seasonality and short life of fresh citrus, extend the citrus industry chain, and meet the nutritional requirements of novel fruit products from consumers. In conclusion, citrus is a functional and nutritional fruit and can complement other fruit and fruit products for various applications.

However, there are some challenges and issues that should be overcome or addressed in the future study of citrus. Under the premise of accurate profiling of phytochemical and nutritional substances in citrus, and given the tremendous differences in the chemical substances among different citrus varieties, fruit parts, maturity, and cultivation region, targeted utilization of citrus resources should be made in the future. Nowadays, citrus industries are confronted with the dilemma that the phytochemicals and nutrients in citrus are vulnerable when introduced into the food and digestive system, hence more studies on the protection of these bioactive compounds are of great importance for enhancing the commercial value of citrus. Moreover, at present, clinical research on citrus mainly focuses on the structure-activity relationships of specific compounds extracted from citrus, while as a whole, the health-promoting effect of citrus on human health should be paid more attention. To meet the growing demand for high-quality citrus products from the market, we should develop novel cultivation strategies and processing techniques to maximally retain citrus flavor, phytochemical, and nutritional values. The health potentials of these novel citrus products also need to be clarified to facilitate their food application. Furthermore, the food applications of citrus are mainly determined by their flavor profiles, the mapping and correlation between citrus sensory quality and specific flavor compounds should also be systematically revealed. From the perspective of sustainable development, the residues of citrus, such as peels, pomace, seeds, and leaves, have a high rate of waste worldwide and are unfavorable for the environment. These side streams in foods and other industries should be exploited as equally important as citrus flesh to broaden their utilization in the future. And environmentally friendly protocols must be preferentially applied during the valorization of citrus.

## Author contributions

SL and YLo reviewed the literature and elaborated the figures. SL, YLo, YLi, and JZ drafted the manuscript. PL and BY: writing—review and editing. QG: writing—review and editing, and funding acquisition. All authors contributed to the article and approved the submitted version.

## Funding

This work was supported by the Chinese Academy of Engineering Academy-Locality Cooperation Project (No. 2019-ZJ-JS-02) and the National Key Research and Development Program of China (No. 2017YFE0122300).

## Conflict of interest

The authors declare that the research was conducted in the absence of any commercial or financial relationships that could be construed as a potential conflict of interest.

## Publisher's note

All claims expressed in this article are solely those of the authors and do not necessarily represent those of their affiliated organizations, or those of the publisher, the editors and the reviewers. Any product that may be evaluated in this article, or claim that may be made by its manufacturer, is not guaranteed or endorsed by the publisher.
